# Disease activity improvements with optimal discriminatory ability between treatment arms: applicability in early and established rheumatoid arthritis clinical trials

**DOI:** 10.1186/s13075-019-2005-9

**Published:** 2019-11-10

**Authors:** Josef Smolen, Roy Fleischmann, Daniel Aletaha, Yihan Li, Yijie Zhou, Iain Sainsbury, Ivan Lagunes Galindo

**Affiliations:** 1 Division of Rheumatology Department of Internal Medicine 3, Medical University of Vienna and Hietzing Hospital, Waehringer Guertel 18-20, A-1090 Vienna, Austria; 2 University of Texas Southwestern, Dallas, TX USA; 3 AbbVie Inc, North Chicago, IL USA; 4 AbbVie Ltd, Cambridge, UK

**Keywords:** Rheumatoid arthritis, Disease activity, Biologicals, Clinical trial

## Abstract

**Background:**

The ACR20 has been validated as the best discriminator of efficacy in placebo-controlled trials, but not in head-to-head trials comparing effective therapies in patients with rheumatoid arthritis (RA). We assessed the most discriminatory ACR response and most discriminatory percent improvement in disease activity measures for Simplified Disease Activity index (SDAI), Clinical Disease Activity index (CDAI), and 28-joint Disease Activity Score based on C-reactive protein (DAS28(CRP)) using different patient populations and trial designs.

**Methods:**

Data from two placebo-controlled studies in established RA and two head-to-head studies in early RA were analyzed. The numeric ACR response for each treatment and *P* value for the difference between treatments were calculated at multiple time points to determine the ACR response associated with the lowest *P* value. Similarly, values for percent improvement from baseline in SDAI, CDAI, and DAS28(CRP) with the most discrimination between treatments were examined.

**Results:**

In the head-to-head early RA trials, the minimum *P* value and greatest treatment difference between the active comparator arms at 6 months was achieved at higher ACR rates and greater percent improvements in other disease activity measures. In established RA, lower responses (minimum *P* value and maximum treatment difference) and smaller improvements in disease activity scores had better discriminatory ability at 6 months.

**Conclusions:**

The most discriminatory ACR response rate and percent improvement in disease activity measures were higher in head-to-head active comparator trials in early RA versus placebo-controlled trials in established RA. This difference should be considered in future clinical trial designs.

**Trial registration:**

NCT00195663, NCT00420927, NCT00195702.

In patients with rheumatoid arthritis (RA), the American College of Rheumatology (ACR) 20 response rate was determined to best discriminate active from placebo (PBO) treatment [1]. The determination of ACR20 as a better discriminator than ACR50 and ACR70 was largely based on evaluation of responses from PBO- or active-controlled trials of conventional synthetic disease-modifying antirheumatic drugs (csDMARDs), including methotrexate (MTX), cyclosporine, and gold injections, in patients with established RA [2]. In the past two decades, much progress has been made in the treatment of RA, with the addition of biological DMARDs (bDMARDs) and alternative csDMARDs to our armamentarium. It has also not been demonstrated that the ACR20 is the best discriminator between treatments in head-to-head studies comparing two active treatments. An alternative, the ACRHybrid measure, was proposed 10 years ago and combines the ACR20, 50, and 70 and a continuous measure of change in the 7 ACR core set measures [3], but it has not been adopted as a primary outcome measure for large-scale RA clinical trials [4]. One objective of this analysis was to determine the ACR response rate that has the most discriminatory ability between different treatment strategies in patients with RA in clinical trials.

When assessing depth of response with respect to disease activity, continuous scales, such as the Simplified Disease Activity index (SDAI), Clinical Disease Activity index (CDAI), or 28-joint Disease Activity Score based on erythrocyte sedimentation rate (DAS28(ESR)) or based on C-reactive protein (DAS28(CRP)) are commonly employed. The DAS28(CRP) is particularly used in clinical trials, which typically include central laboratory testing. Another objective of this analysis was to evaluate what criteria based on improvements in these disease activity indices most discriminate between different treatment strategies in clinical trials.

## Patients and methods

### Patients and studies

This post hoc analysis included data from patients from four double-blind, randomized, PBO- or active-controlled trials of originator adalimumab (ADA) in patients with RA. The individual studies were performed in accordance with the International Conference on Harmonisation Guidelines for Good Clinical Practice and the Declaration of Helsinki.

The individual study protocols were reviewed and approved by an institutional review board or ethics committee at each study center. All patients provided written informed consent.

The OPTIMA and PREMIER studies compared active treatments with initial therapy of ADA+MTX versus MTX in patients with early RA, whereas the ARMADA and DE019 studies compared treatment with ADA versus PBO, both added to background methotrexate in patients with established RA. The methods of these trials have been previously published [5–8]. Briefly, in the phase 3 PREMIER trial (NCT00195663), patients with early RA who were naïve to tumor necrosis factor inhibitors (TNFi) and MTX, were blindly randomized to receive ADA 40 mg every other week (eow), MTX weekly, or ADA 40 mg eow plus MTX weekly for 2 years [5]. In the phase 4 OPTIMA trial (NCT00420927), patients with early RA who were naïve to MTX and TNFi were blindly randomized to receive ADA 40 mg eow plus MTX weekly or PBO plus MTX for 26 weeks followed by a 52-week treatment continuation, adjustment, or withdrawal period [6].

In the phase 3 DE019 trial (NCT00195702), TNFi-naïve patients with established RA and active disease despite treatment with MTX were randomized to receive ADA 20 mg eow, ADA 40 mg eow, or PBO in addition to background MTX for 1 year [7]. In ARMADA (conducted before the requirement of clinical trial registration), patients with established RA with an inadequate response to ≤ 4 prior csDMARD(s) were randomized to receive ADA at 20, 40, or 80 mg eow or PBO, each with concomitant MTX for 24 weeks [8].

### Identification of the most discriminatory ACR response, SDAI, CDAI, and DAS28(CRP) improvement

With adalimumab being an established therapy to treat RA, we defined the criteria for “most discriminatory” as the ability to determine a statistically significant difference between adalimumab and the appropriate control. The response that corresponds to the lowest *P* value is therefore identified as the most discriminatory, with the consideration that the lowest *P* value is equivalent to the largest standardized effect size in a completed trial with fixed sample size.

For each treatment, the ACR response in increments of 5% (0–100%) was calculated at 12 and 24/26 weeks. The ACR response which corresponded to the lowest *P* value for the difference between the ADA+MTX versus PBO+MTX in early RA and ADA versus PBO+background MTX in established RA was identified as having the most discriminatory ability. In addition, the ACR response with the greatest treatment difference was also investigated.

Following a similar reasoning, percent improvement in SDAI, CDAI, and DAS28(CRP) scores (in 5% increments) with the most discriminatory ability between treatments, or between active treatment and PBO was identified. The percent improvement which corresponded to the lowest *P* value for the between-treatment difference was identified as having the most discriminatory activity. The percent improvement with the greatest treatment difference was also investigated. Subgroup analyses of CDAI response based on baseline CDAI and baseline DAS28(CRP) (≤ median vs > median) were also conducted to assess the impact of baseline disease activity on the most discriminatory cutoff.

## Results

From the early RA studies, 268 (PREMIER) and 515 (OPTIMA) patients receiving newly initiated ADA+MTX and 257 (PREMIER) and 517 (OPTIMA) patients receiving newly initiated PBO+MTX were included in this analysis. From the established RA studies with continued background MTX, 207 (DE019) and 67 (ARMADA) patients receiving ADA+MTX and 200 (DE019) and 62 (ARMADA) patients receiving PBO+MTX were included in this analysis. Baseline characteristics were generally similar between the two treatment groups in both early RA and established RA patients, although differences were observed between studies (Table 1).

**Table 1:** Baseline characteristics

Mean ± SD	PREMIER		OPTIMA	
	ADA+MTX (*n* = 268)	PBO+MTX (*n* = 257)	ADA+MTX (*n* = 515)	PBO+MTX (*n* = 517)
Age, years	51.9 ± 14.0	52.0 ± 13.1	50.7 ± 14.5	50.4 ± 13.6
Female, *n* (%)	193 (72.0)	190 (73.9)	380 (73.8)	382 (73.9)
Caucasian, *n* (%)	250 (93.3)	242 (94.2)	460 (89.3)	464 (89.7)
Duration of RA, years	0.7 ± 0.8	0.8 ± 0.9	0.3 ± 0.3	0.4 ± 0.6
CRP, mg/dL	3.9 ± 4.2	4.0 ± 4.0	2.7 ± 3.2	3.0 ± 3.3
DAS28(CRP)	6.3 ± 0.9	6.3 ± 0.9	6.0 ± 1.0	6.0 ± 1.0
CDAI	43.6 ± 12.6	44.4 ± 11.9	41.3 ± 13.2	40.3 ± 13.3
SDAI	47.4 ± 14.2	48.4 ± 13.4	44.0 ± 14.2	43.2 ± 14.6
TJC68	30.7 ± 14.2	32.3 ± 14.3	29.0 ± 15.0	27.2 ± 14.5
SJC66	21.1 ± 11.2	22.1 ± 11.7	18.3 ± 10.5	17.9 ± 10.5
PGA	65.1 ± 17.6	65.6 ± 17.7	63.0 ± 17.8	62.2 ± 18.3
PtGA	66.8 ± 22.1	63.0 ± 25.0	64.0 ± 23.0	62.5 ± 22.1
Pt pain	62.5 ± 21.3	59.6 ± 24.3	65.0 ± 21.3	64.7 ± 21.2
HAQ-DI	1.5 ± 0.6	1.5 ± 0.6	1.6 ± 0.7	1.6 ± 0.7
	DE019		ARMADA	
	ADA+MTX (*n* = 207)	PBO+MTX (*n* = 200)	ADA+MTX (*n* = 67)	PBO+MTX (*n* = 62)
Age, years	56.1 ± 13.5	56.1 ± 12.0	57.2 ± 11.4	56.0 ± 10.8
Female, *n* (%)	158 (76.3)	146 (73.0)	50 (74.5)	51 (82.3)
Caucasian, *n* (%)	173 (83.6)	166 (83.0)	58 (86.6)	45 (72.6)
Duration of RA, years	11.0 ± 9.2	10.9 ± 8.8	12.2 ± 11.1	11.1 ± 8.0
CRP, mg/dL	1.8 ± 2.3	1.8 ± 2.1	2.1 ± 1.8	3.1 ± 3.9
DAS28(CRP)	5.7 ± 0.9	5.8 ± 0.9	5.1 ± 0.7	5.1 ± 1.0
CDAI	38.6 ± 12.2	40.3 ± 12.3	37.3 ± 10.7	37.3 ± 11.7
SDAI	40.6 ± 13.0	42.1 ± 12.9	39.5 ± 10.7	40.4 ± 13.5
TJC68	27.3 ± 12.7	28.1 ± 13.8	28.0 ± 12.7	28.7 ± 15.2
SJC66	19.3 ± 9.8	19.0 ± 9.5	17.3 ± 8.6	16.9 ± 9.5
PGA	62.0 ± 16.7	61.3 ± 17.3	58.7 ± 15.8	58.9 ± 15.3
PtGA	52.7 ± 21.0	54.3 ± 22.9	56.9 ± 21.1	58.0 ± 23.2
Pt pain	55.9 ± 20.4	56.3 ± 22.9	53.0 ± 22.0	57.2 ± 21.0
HAQ-DI	1.5 ± 0.6	1.5 ± 0.6	1.6 ± 0.6	1.6 ± 0.6

*ADA* adalimumab, *CDAI* Clinical Disease Activity index, *CRP* C-reactive protein, *DAS28* Disease Activity Score based on 28-joints, *HAQ-DI* Health Assessment Questionnaire Disability Index, *PBO* placebo, *PGA* Physician Global Assessment, *Pt* patient, *PtGA* Patient Global Assessment, *RA* rheumatoid arthritis, *SDAI* Simplified Disease Activity index, *SJC66* swollen joint count based on 66 joints, *TJC68* tender joint count based on 68 joints

### Identification of the most discriminatory ACR response

**Fig. 1:**
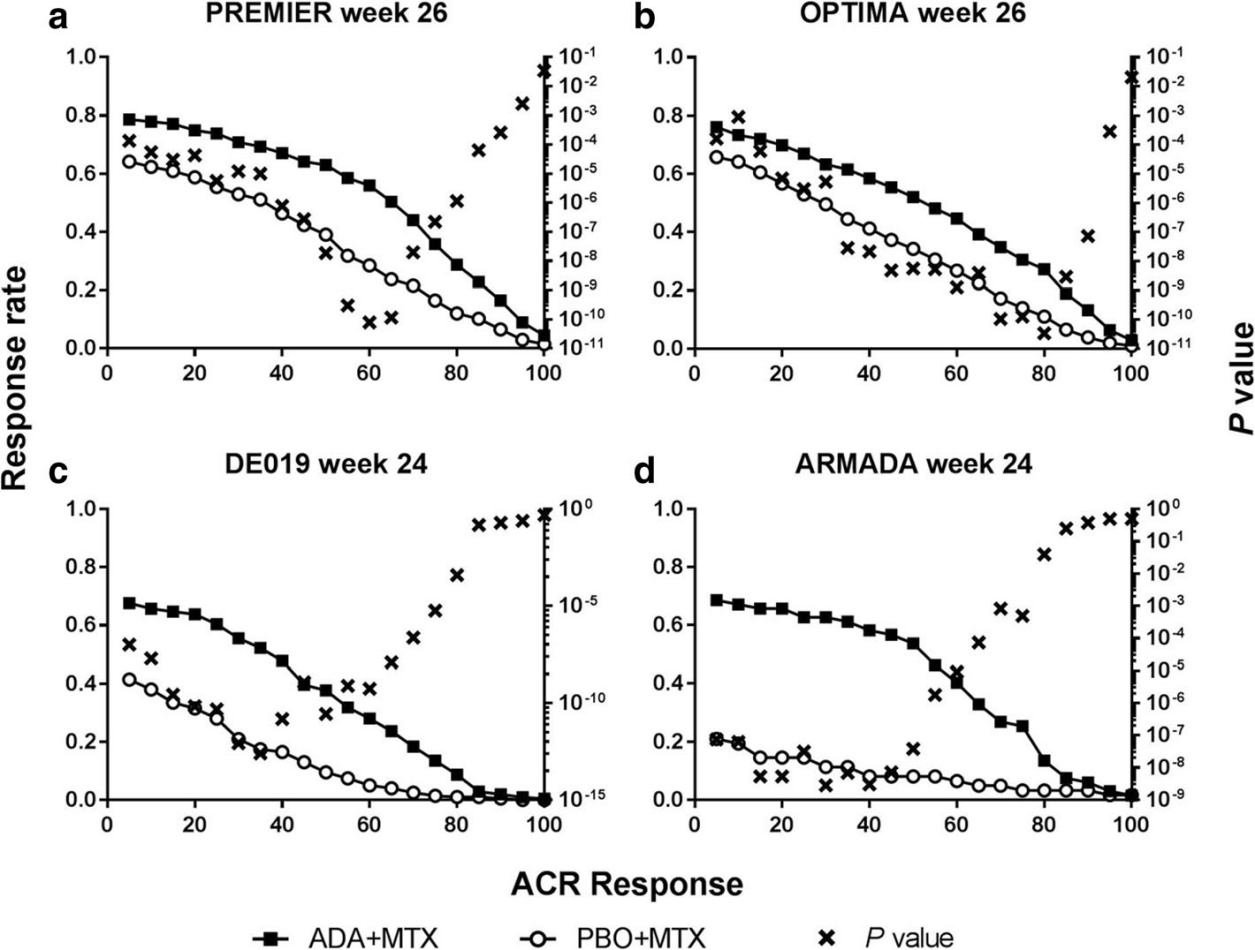
ACR responses in patients with early RA **a** PREMIER and **b** OPTIMA, or established RA **c** DE019 and **d** ARMADA at week 24/26. *P* value for difference between response rates for patients treated with ADA+MTX and PBO+MTX. ADA, adalimumab; ACR, American College of Rheumatology; MTX, methotrexate; PBO, placebo; RA, rheumatoid arthritis

In patients with early RA from PREMIER who were initiating MTX, the lowest *P* value for the difference in ACR responses between the PBO+MTX arm and the ADA+MTX arm and the greatest treatment difference was achieved at ACR60 (Fig. 1a) at week 26. In patients with early RA from OPTIMA who were initiating MTX, the most discrimination at week 26 in terms of the lowest *P* value was achieved at ACR80, while similar absolute treatment differences were observed between ACR35–80 (Fig. 1b and Additional file 1: Table S1). The optimal ACR responses for both trials were lower at week 12 (ACR30 for PREMIER and ACR55–60 for OPTIMA (see Additional file 1: Figure S1). Conversely, for patients with established RA from DE019 and ARMADA who were continuing background MTX, lower ACR responses for both the lowest *P* value and the greatest treatment difference were more discriminatory when comparing PBO+MTX versus ADA+MTX; ACR35 and ACR30 for DE019 and ARMADA respectively, at week 24 (Fig. 1c, d and Additional file 1: Table S1). At week 12, ACR10 was most sensitive to differences between treatments (see Additional file 1: Figure S1). When considering the standard ACR cutoff points, ACR50/70 were more discriminatory for early RA and ACR20 for established RA at week 24/26 in this analysis.

### Identification of the most discriminatory CDAI and SDAI improvements

**Fig. 2:**
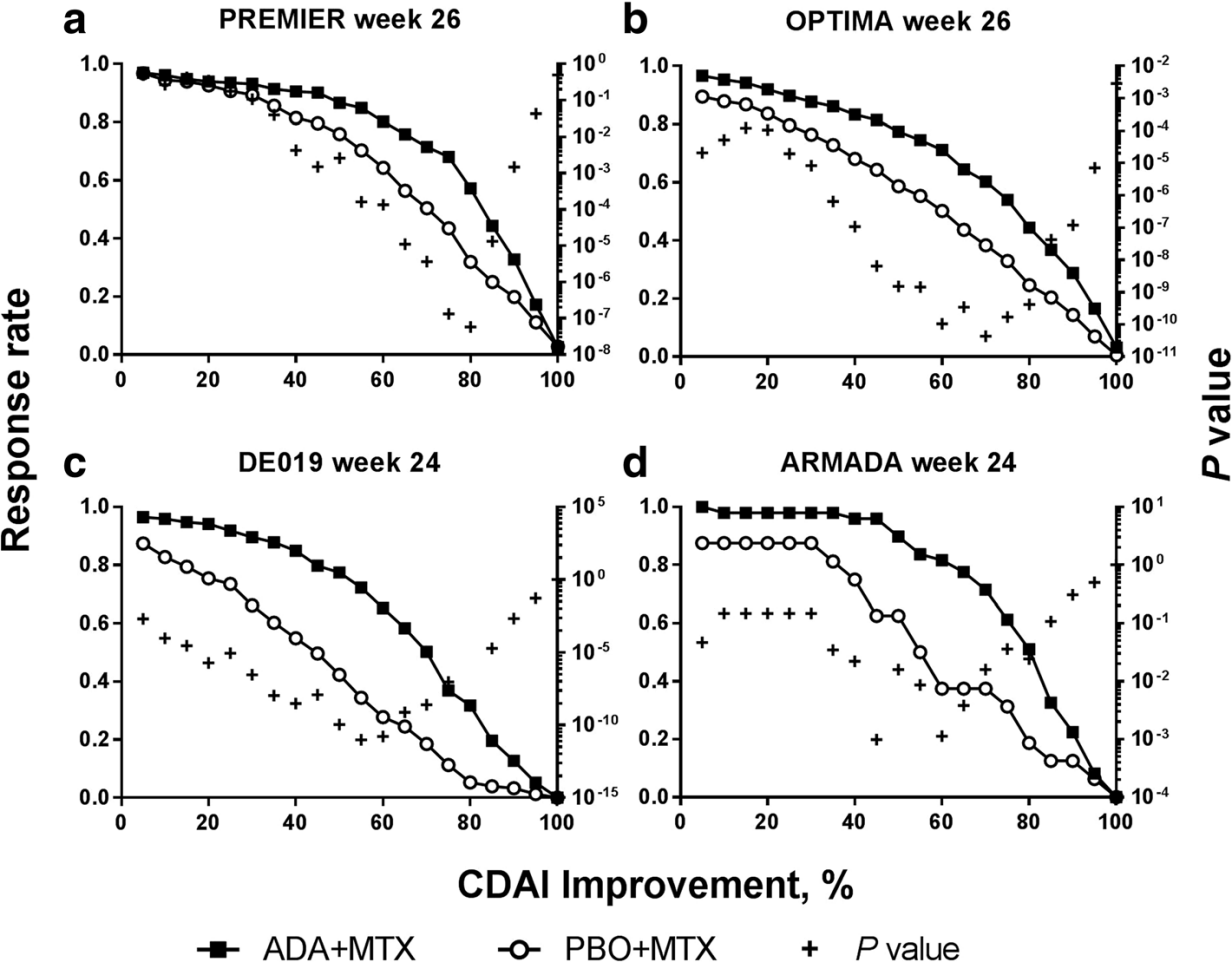
Percent change from baseline in CDAI in patients with early RA from **a** PREMIER and **b** OPTIMA, or established RA from **c** DE019 and **d** ARMADA at week 24/26. *P* value for difference between response rates for patients treated with ADA+MTX and PBO+MTX. ADA, adalimumab; CDAI, Clinical Disease Activity index; MTX, methotrexate; PBO, placebo; RA, rheumatoid arthritis

In early RA patients from PREMIER and OPTIMA, the most discrimination in terms of both the lowest *P* value and the greatest difference between the PBO+MTX arm versus the ADA+MTX arm at week 26 was achieved with CDAI 80% and CDAI 70%, respectively (Fig. 2a, b and Additional file 1: Table S1). For both trials, the CDAI improvement with the most discrimination at week 12 was consistent with that for week 24 (CDAI 65–80%) for PREMIER and CDAI 70–80% for OPTIMA (see Additional file 1: Figure S2). In patients with established RA from the DE019 trial, CDAI 55% had the most discriminatory ability in terms of both the lowest *P* value and the greatest treatment difference at week 24, when comparing PBO+MTX versus ADA+MTX (Fig. 2c and Additional file 1: Table S1). At week 12, however, the most discrimination was observed at CDAI 40% (see Additional file 1: Figure S2). In established RA patients from the ARMADA trial, the lowest *P* value comparing ADA+MTX versus PBO+MTX at week 24 was observed at CDAI 45%, while the greatest difference was observed at CDAI 60% (Fig. 2d and Additional file 1: Table S1). At week 12, the greatest difference and lowest *P* value between treatment arms was observed at CDAI 50%. When considering CDAI improvement cutoffs identified previously [9], CDAI 70% or 85% was more discriminatory for early RA and CDAI 50% for established RA at week 24/26.

In the CDAI subgroup analyses based on baseline CDAI and DAS28(CRP) subgroups (≤ median vs > median), the results were generally consistent with the overall population and between the subgroups (Additional file 1: Table S2).

**Fig. 3:**
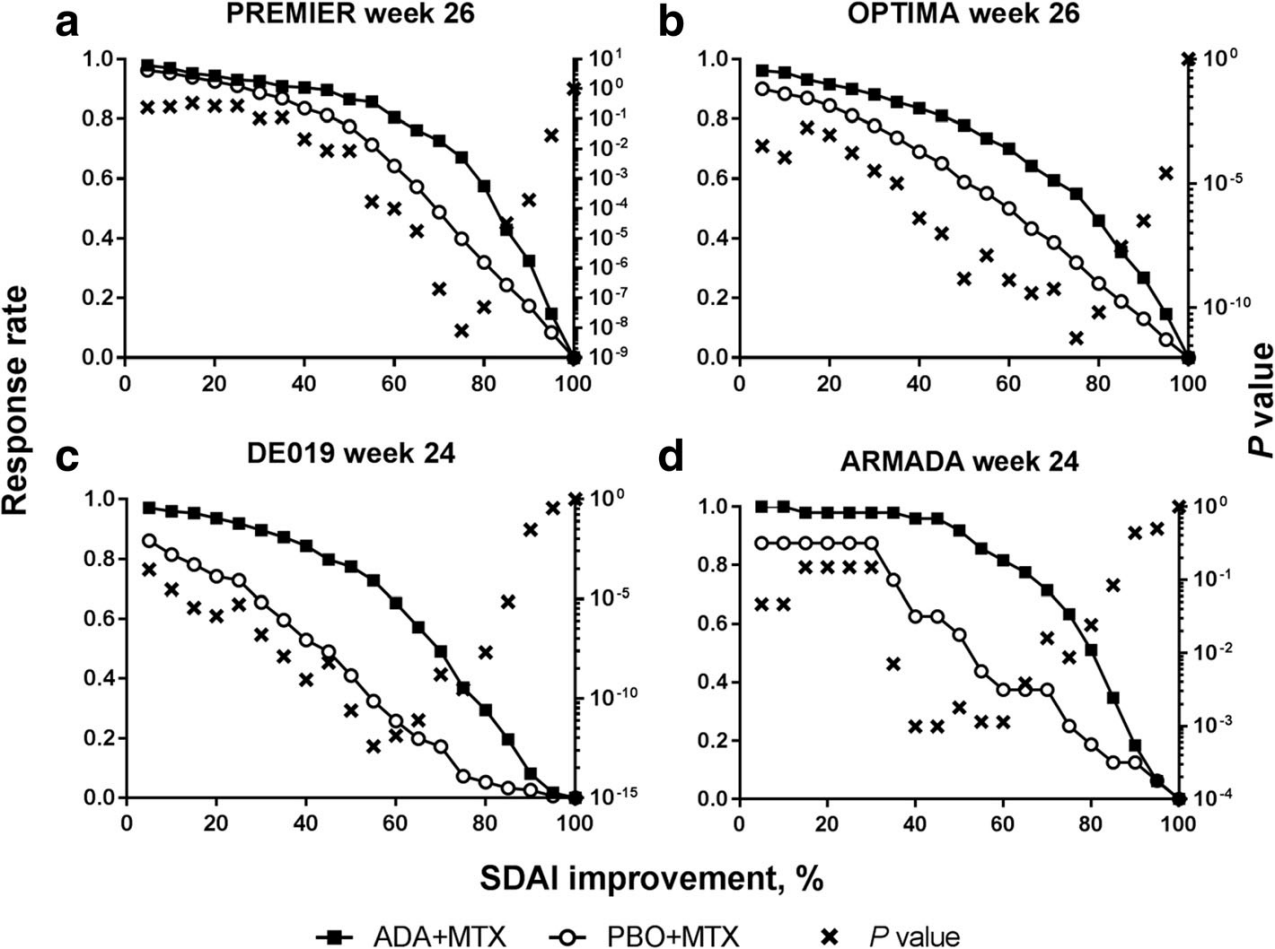
Percent change from baseline in SDAI in patients with early RA from **a** PREMIER and **b** OPTIMA, or established RA from **c** DE019 and **d** ARMADA at week 24/26. *P* value for difference between response rates for patients treated with ADA+MTX and PBO+MTX. ADA, adalimumab; MTX, methotrexate; PBO, placebo; RA, rheumatoid arthritis; SDAI, Simplified Disease Activity Index

Similar to the observations for CDAI, 75% improvement in SDAI had the most discriminatory ability for both the lowest *P* value and the greatest treatment difference between ADA+MTX and PBO+MTX treatment for patients in early RA trials at week 26 (Fig. 3a, b and Additional file 1: Table S1); at week 12, the highest discriminatory ability was between SDAI 70–80% for both trials. In established RA patients in DE019, SDAI 55% (Fig. 3c) had the most discriminatory ability for both the lowest *P* value and the greatest treatment difference at week 24. At week 24 in ARMADA, the lowest *P* value was observed at SDAI 40–45%, while the greatest difference was observed at SDAI 60% (Fig. 3d and Additional file 1: Table S1). At week 12, the lowest *P* value and the greatest difference between treatment arms were observed at SDAI 45–50% in both trials (see Additional file 1: Figure S3). Consistent with CDAI, when considering SDAI improvement cutoffs identified previously [9], SDAI 70% or 85% was more discriminatory for early RA and SDAI 50% for established RA at week 24/26.

### Identification of the most discriminatory DAS28(CRP) improvements

**Fig. 4:**
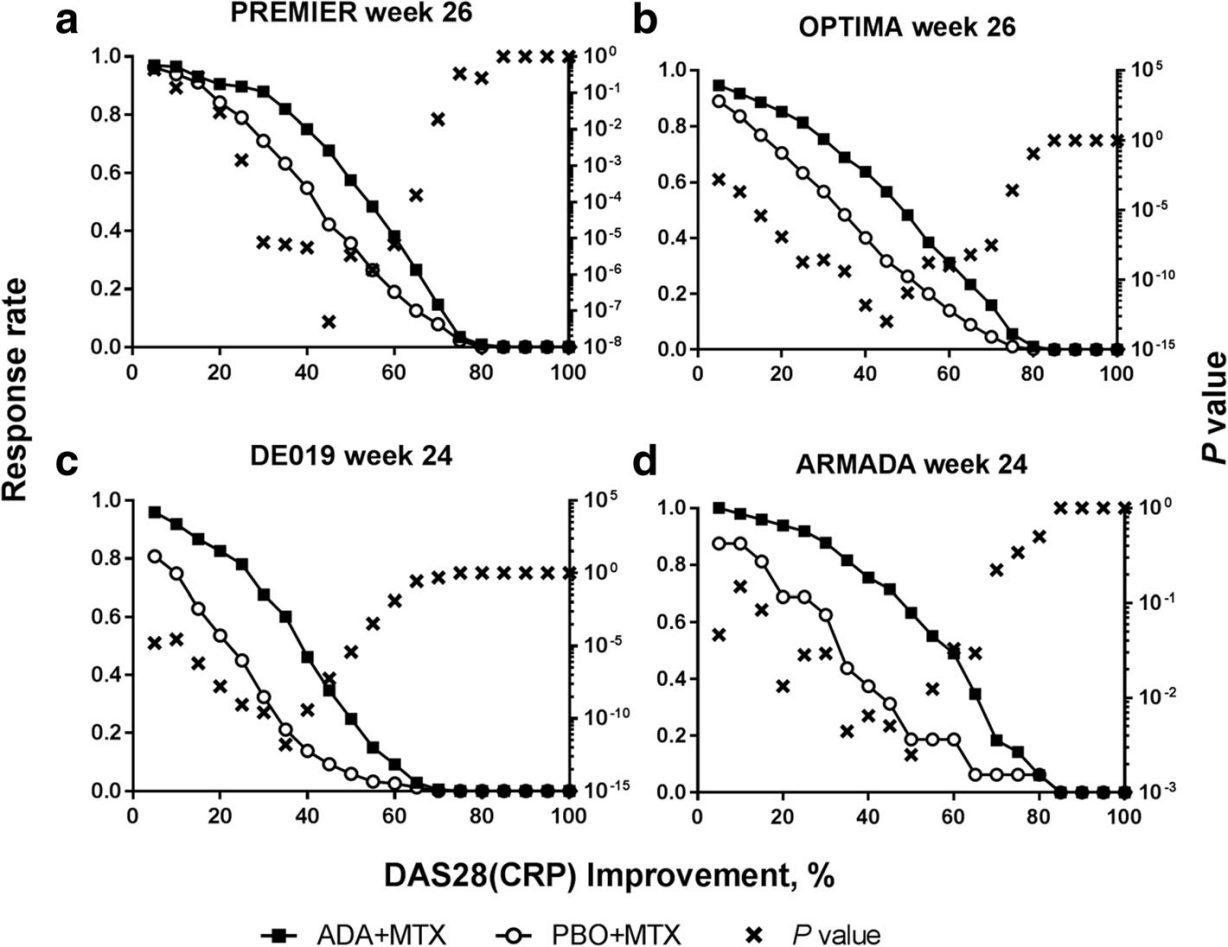
Percent change from baseline in DAS28(CRP) in patients with early RA from **a** PREMIER and **b** OPTIMA, or established RA from **c** DE019 and **d** ARMADA at week 24/26. *P* value for difference between response rates for patients treated with ADA+MTX and PBO+MTX. ADA, adalimumab; DAS28(CRP), 28-joint Disease Activity Score based on C-reactive protein; MTX, methotrexate; PBO, placebo; RA, rheumatoid arthritis

For both trials in early RA patients, an improvement in DAS28(CRP) of 45% had the most discriminatory ability for both the lowest *P* value and greatest difference between the ADA+MTX and PBO+MTX at week 24 (Fig. 4a, b and Additional file 1: Table S1). Consistently, the most discriminatory percent improvement at week 12 was also between DAS28(CRP) 40–45% for both trials. At week 24, in patients with established RA, the most discrimination for both the lowest *P* value and the greatest treatment difference was observed at DAS28(CRP) 35% in DE019 and DAS28(CRP) 50% in ARMADA (Fig. 4c, d and Additional file 1: Table S1). For both trials at week 12, the most discriminatory percent improvements tended to be lower, between DAS28(CRP) 5–25% (see Additional file 1: Figure S4).

## Discussion

We performed a comprehensive analysis to identify the ACR response and percent improvement from baseline in commonly used disease activity measures that would provide the most discrimination between treatment arms in clinical trials. In patients with established RA with inadequate response to MTX, when comparing an active agent versus PBO (plus background MTX), lower ACR response and smaller percent improvements from baseline in disease activity measures (CDAI, SDAI, and DAS28(CRP)) had better discriminatory ability. Conversely, in patients with early RA, when comparing two active agents, higher ACR responses and greater percent improvements from baseline in disease activity measures had better discriminatory ability. This suggests that the best separation is by depth of response because two active medications are likely to perform similarly in terms of ACR20 response whereas the true difference in efficacy should be observed with higher ACR response. Our results are consistent with an earlier report, which demonstrated that greater SDAI improvements and higher ACR responses were more discriminatory in an early RA population, while smaller improvements and lower degrees of improvement showed better discrimination in an established RA population [10]. We observed greater consistency in the discriminatory performance of CDAI and SDAI improvements as compared with that of ACR scores and DAS28(CRP) across trials and time points with respect to a similar disease population (Additional file 1: Table S1).

Our findings confirm the original conclusions of Felson et al., who demonstrated that the ACR20 response is superior in differentiating an active treatment from a PBO [2]. The ACR20 response is the “gold standard” in RA clinical trials to differentiate active from PBO response or to compare different treatments [1]. In several recent trials, a high ACR20 response was observed with PBO as an add-on to background MTX in patients with active RA, which may be due to differences in patient populations studied (e.g., geographic regions), differences in pre-study therapy, enhanced clinical management compared with standard of care, or differences in therapies studied, rather than a consequence of using the measure itself [9, 11–13].

Our analysis included both early and established RA populations treated with ADA and MTX or PBO and MTX. The patients in the early RA trials were MTX-naïve and were initiating MTX, so the comparison was between two active treatments. On the other hand, patients in the established RA trials included patients with active RA despite MTX treatment who were initiating ADA or PBO on background MTX. This most likely contributed to the different findings between these two populations in the most discriminatory response cutoffs and improvements. The small difference between OPTIMA and PREMIER may relate to the longer mean disease duration in PREMIER.

In general, greater improvements became more discriminatory after 6 months (24/26 weeks) of treatment compared with 12 weeks of treatment, indicating that the depth of treatment response improved over time. As expected, the improvements in CDAI and SDAI with the optimal discriminatory activity between treatments were very similar. The most discriminatory improvements in DAS28(CRP) for early RA tended to be much lower (45%) compared with the CDAI and SDAI improvements (70–80%). This is consistent with the weighting and transformation associated with the DAS28(CRP) formula, which results in a lack of linearity with increasing activity. Importantly, the optimally discriminatory improvements in SDAI and CDAI tended to be more consistent between trials in the same type of patients than the optimal ACR scores (e.g., SDAI 75% improvement for both OPTIMA and PREMIER at week 26 vs ACR60 and ACR80, respectively). In addition, at early time points (12 weeks), the optimally discriminatory ACR improvement identified for early RA (ACR10) may not be as clinically meaningful as SDAI and CDAI improvements, which tended to be higher.

Limitations of this analysis include the lack of data to compare two active treatments in a head-to-head study of bDMARDs or csDMARDs in patients with established RA. However, this was done in the recent ORAL Strategy trial, which was a head-to-head comparison of tofacitinib monotherapy, tofacitinib+MTX, and ADA+MTX in patients with active RA despite MTX therapy and used ACR50 as the primary endpoint [14]. Additionally, adalimumab has been compared with other bDMARDs in head-to-head trials with similar results [15–17], suggesting that the data are generalizable. Moreover, the results are further indirectly supported by the fact that agents that directly interfere with interleukin-6 pathways, and thus reduce CRP or ESR irrespective of clinical improvement, exaggerate DAS28 responses because of the high weight of CRP/ESR in the DAS28 formula [18, 19].

## Conclusions

In conclusion, our post hoc analysis suggests that different optimal ACR responses or improvements in disease activity measures may have to be used in trials in early and established RA patients, or when comparing a drug with an active or a PBO comparator. Moreover, it appears that although the ACR scores and DAS28(CRP) are commonly used, they did not perform as consistently for discriminatory purposes as measures developed more recently, such as SDAI and CDAI. These measures therefore may be potentially considered for future trials. Finding a consensus for the use of these different response criteria may be a task for the future.

## Supplementary information

**Additional file 1:** **Table S1.** Most discriminatory ACR, CDAI, SDAI and DAS28(CRP) cutoffs for each trial. **Table S2.** Most discriminatory CDAI cutoffs at week 24/26 for each trial in all patients and in subgroups of patients based on baseline CDAI and DAS28(CRP). **Figure S1.** ACR response rate in patients with early RA from (A) PREMIER and (B) OPTIMA and in patients with established RA from (C) DE019 and (D) ARMADA at week 12. *P* value of difference between response rates for patients treated with ADA+MTX and PBO+MTX. ADA, adalimumab; ACR, American College of Rheumatology; MTX, methotrexate; PBO, placebo; RA, rheumatoid arthritis. **Figure S2.** Percent change from baseline in CDAI scores in patients with early RA from (A) PREMIER and (B) OPTIMA and in patients with established RA from (C) DE019 and (D) ARMADA at week 12. *P* value of difference between response rates for patients treated with ADA+MTX and PBO+MTX. ADA, adalimumab; CDAI, Clinical Disease Activity Index; MTX, methotrexate; PBO, placebo; RA, rheumatoid arthritis. **Figure S3.** Percent change from baseline in SDAI scores in patients with early RA from (A) PREMIER and (B) OPTIMA and in patients with established RA from (C) DE019 and (D) ARMADA at week 12. P value of difference between response rates for patients treated with ADA+MTX and PBO+MTX. ADA, adalimumab; MTX, methotrexate; PBO, placebo; RA, rheumatoid arthritis; SDAI, Simplified Disease Activity Index. **Figure S4.** Percent change from baseline in DAS28(CRP) scores in patients with early RA from (A) PREMIER and (B) OPTIMA and in patients with established RA from (C) DE019 and (D) ARMADA at week 12. *P* value of difference between response rates for patients treated with ADA+MTX and PBO+MTX. ADA, adalimumab; DAS28(CRP), 28-joint Disease Activity Score based on C-reactive protein; MTX, methotrexate; PBO, placebo; RA, rheumatoid arthritis.

## Data Availability

AbbVie is committed to responsible data sharing regarding the clinical trials we sponsor. This includes access to anonymized, individual and trial-level data (analysis data sets), as well as other information (e.g., protocols and Clinical Study Reports), as long as the trials are not part of an ongoing or planned regulatory submission. This includes requests for clinical trial data for unlicensed products and indications. This clinical trial data can be requested by any qualified researchers who engage in rigorous, independent scientific research, and will be provided following review and approval of a research proposal and Statistical Analysis Plan (SAP) and execution of a Data Sharing Agreement (DSA). Data requests can be submitted at any time and the data will be accessible for 12 months, with possible extensions considered. For more information on the process, or to submit a request, visit the following link: https://www.abbvie.com/our-science/clinical-trials/clinical-trials-data-and-information-sharing/data-and-information-sharing-with-qualified-researchers.html.

## References

[1] Felson DT, Anderson JJ, Boers M, Bombardier C, Furst D, Goldsmith C, Katz LM, Lightfoot R, Paulus H, Strand V (1995). American College of Rheumatology. Preliminary definition of improvement in rheumatoid arthritis. Arthritis Rheum.

[2] Felson DT, Anderson JJ, Lange ML, Wells G, LaValley MP (1998). Should improvement in rheumatoid arthritis clinical trials be defined as fifty percent or seventy percent improvement in core set measures, rather than twenty percent?. Arthritis Rheum.

[3] American College of Rheumatology Committee to Reevaluate Improvement C (2007). A proposed revision to the ACR20: the hybrid measure of American College of Rheumatology response. Arthritis Rheum.

[4] Felson DT, LaValley MP (2014). The ACR20 and defining a threshold for response in rheumatic diseases: too much of a good thing. Arthritis Res Ther.

[5] Breedveld FC, Weisman MH, Kavanaugh AF, Cohen SB, Pavelka K, van Vollenhoven R, Sharp J, Perez JL, Spencer-Green GT (2006). The PREMIER study: a multicenter, randomized, double-blind clinical trial of combination therapy with adalimumab plus methotrexate versus methotrexate alone or adalimumab alone in patients with early, aggressive rheumatoid arthritis who had not had previous methotrexate treatment. Arthritis Rheum.

[6] Smolen JS, Emery P, Fleischmann R, van Vollenhoven RF, Pavelka K, Durez P, Guerette B, Kupper H, Redden L, Arora V (2014). Adjustment of therapy in rheumatoid arthritis on the basis of achievement of stable low disease activity with adalimumab plus methotrexate or methotrexate alone: the randomised controlled OPTIMA trial. Lancet.

[7] Keystone EC, Kavanaugh AF, Sharp JT, Tannenbaum H, Hua Y, Teoh LS, Fischkoff SA, Chartash EK (2004). Radiographic, clinical, and functional outcomes of treatment with adalimumab (a human anti-tumor necrosis factor monoclonal antibody) in patients with active rheumatoid arthritis receiving concomitant methotrexate therapy: a randomized, placebo-controlled, 52-week trial. Arthritis Rheum.

[8] Weinblatt ME, Keystone EC, Furst DE, Moreland LW, Weisman MH, Birbara CA, Teoh LA, Fischkoff SA, Chartash EK (2003). Adalimumab, a fully human anti–tumor necrosis factor α monoclonal antibody, for the treatment of rheumatoid arthritis in patients taking concomitant methotrexate: the ARMADA trial. Arthritis Rheum.

[9] Keystone EC, Taylor PC, Drescher E, Schlichting DE, Beattie SD, Berclaz PY, Lee CH, Fidelus-Gort RK, Luchi ME, Rooney TP (2015). Safety and efficacy of baricitinib at 24 weeks in patients with rheumatoid arthritis who have had an inadequate response to methotrexate. Ann Rheum Dis.

[10] Aletaha D, Martinez-Avila J, Kvien TK, Smolen JS (2012). Definition of treatment response in rheumatoid arthritis based on the simplified and the clinical disease activity index. Ann Rheum Dis.

[11] Kivitz AJ, Gutierrez-Urena SR, Poiley J, Genovese MC, Kristy R, Shay K, Wang X, Garg JP, Zubrzycka-Sienkiewicz A (2017). Peficitinib, a JAK inhibitor, in the treatment of moderate-to-severe rheumatoid arthritis in patients with an inadequate response to methotrexate. Arthritis Rheumatol.

[12] Huizinga TW, Fleischmann RM, Jasson M, Radin AR, van Adelsberg J, Fiore S, Huang X, Yancopoulos GD, Stahl N, Genovese MC (2014). Sarilumab, a fully human monoclonal antibody against IL-6Ralpha in patients with rheumatoid arthritis and an inadequate response to methotrexate: efficacy and safety results from the randomised SARIL-RA-MOBILITY Part A trial. Ann Rheum Dis.

[13] Westhovens R, Taylor PC, Alten R, Pavlova D, Enriquez-Sosa F, Mazur M, Greenwald M, Van der Aa A, Vanhoutte F, Tasset C (2017). Filgotinib (GLPG0634/GS-6034), an oral JAK1 selective inhibitor, is effective in combination with methotrexate (MTX) in patients with active rheumatoid arthritis and insufficient response to MTX: results from a randomised, dose-finding study (DARWIN 1). Ann Rheum Dis.

[14] Fleischmann R, Mysler E, Hall S, Kivitz AJ, Moots RJ, Luo Z, DeMasi R, Soma K, Zhang R, Takiya L, et al. Efficacy and safety of tofacitinib monotherapy, tofacitinib with methotrexate, and adalimumab with methotrexate in patients with rheumatoid arthritis (ORAL strategy): a phase 3b/4, double-blind, head-to-head, randomised controlled trial. Lancet. 2017;390(10093):457–68. 10.1016/S0140-6736(17)31618-5 28629665

[15] Schiff M, Weinblatt ME, Valente R, van der Heijde D, Citera G, Elegbe A, Maldonado M, Fleischmann R (2014). Head-to-head comparison of subcutaneous abatacept versus adalimumab for rheumatoid arthritis: two-year efficacy and safety findings from AMPLE trial. Ann Rheum Dis.

[16] Smolen JS, Burmester GR, Combe B, Curtis JR, Hall S, Haraoui B, van Vollenhoven R, Cioffi C, Ecoffet C, Gervitz L (2016). Head-to-head comparison of certolizumab pegol versus adalimumab in rheumatoid arthritis: 2-year efficacy and safety results from the randomised EXXELERATE study. Lancet.

[17] Porter D, van Melckebeke J, Dale J, Messow CM, McConnachie A, Walker A, Munro R, McLaren J, McRorie E, Packham J (2016). Tumour necrosis factor inhibition versus rituximab for patients with rheumatoid arthritis who require biological treatment (ORBIT): an open-label, randomised controlled, non-inferiority, trial. Lancet.

[18] Smolen JS, Aletaha D, Gruben D, Zwillich SH, Krishnaswami S, Mebus C (2017). Brief report: remission rates with tofacitinib treatment in rheumatoid arthritis: a comparison of various remission criteria. Arthritis Rheumatol.

[19] Smolen JS, Aletaha D (2011). Interleukin-6 receptor inhibition with tocilizumab and attainment of disease remission in rheumatoid arthritis: the role of acute-phase reactants. Arthritis Rheum.

