# Supplementary material for: Disease activity improvements with optimal discriminatory ability between treatment arms: applicability in early and established rheumatoid arthritis clinical trials
Source: Arthritis Res Ther. 2019 Nov 10;21:231. doi: 10.1186/s13075-019-2005-9 (PMC6842479; doi:10.1186/s13075-019-2005-9)

Additional file 1

Supplemental Table 1. Most discriminatory ACR, CDAI, SDAI and DAS28(CRP) cutoffs for each trial.

	PREMIER		OPTIMA		DE019		ARMADA	
	Lowest *P* value	Greatest Treatment Difference	Lowest *P* value	Greatest Treatment Difference	Lowest *P* value	Greatest Treatment Difference	Lowest *P* value	Greatest Treatment Difference
**Week 24/26**								
ACR	60%	60%	80%	45%	35%	35%	30%	20%
CDAI	80%	80%	70%	70%	55%	55%	45%	60%
SDAI	75%	75%	75%	75%	55%	55%	40–45%	60%
DAS28(CRP)	45%	45%	45%	45%	35%	35%	50%	50%
**Week 12**								
ACR	30%	30%	60%	55%	10%	10%	10%	10%
CDAI	80%	65%	80%	70%	40%	40%	50%	50%
SDAI	70%	70%	80%	80%	45%	45%	50%	50%
DAS28(CRP)	45%	40%	45%	45%	5%	25%	15%	15%

ACR, American College of Rheumatology; CDAI, Clinical Disease Activity Index; CRP, C-reactive protein; DAS28, Disease Activity Score based on 28-joints; SDAI, Simplified Disease Activity Index.

Supplemental Table 2. Most discriminatory CDAI cutoffs at week 24/26 for each trial in all patients and in subgroups of patients based on baseline CDAI and DAS28(CRP)

	PREMIER		OPTIMA		DE019		ARMADA	
	**Lowest P value**	**Greatest Treatment Difference**	**Lowest P value**	**Greatest Treatment Difference**	**Lowest P value**	**Greatest Treatment Difference**	**Lowest P value**	**Greatest Treatment Difference**
Analysis based on all patients								
	80%	80%	70%	70%	55%	55%	45%	60%
Analysis based on baseline CDAI subgroups*								
≤median<br>>median	80%<br>80%	80%<br>80%	60%<br>70%	60%<br>70%	80%<br>60%	55%<br>60%	45%<br>60–65%	45%<br>60–65%
Analysis based on baseline DAS28 subgroups†								
≤median<br>>median	80%<br>80%	80%<br>80%	60%<br>80%	60%<br>70%	80%<br>60%	55%<br>60%	45%<br>60–65%	45%<br>60–65%

*Baseline CDAI median in PREMIER=43.5, OPTIMA=39.0, DE019=37.5, and ARMADA=37.3.

†Baseline DAS28(CRP) median in PREMIER=6.35, OPTIMA=5.97, DE019=5.68, and ARMADA=5.14.

Supplemental Figure 1. ACR response rate in patients with early RA from (A) PREMIER and (B) OPTIMA and in patients with established RA from (C) DE019 and (D) ARMADA at week 12. *P* value of difference between response rates for patients treated with ADA+MTX and PBO+MTX. ADA, adalimumab; ACR, American College of Rheumatology; MTX, methotrexate; PBO, placebo; RA, rheumatoid arthritis.

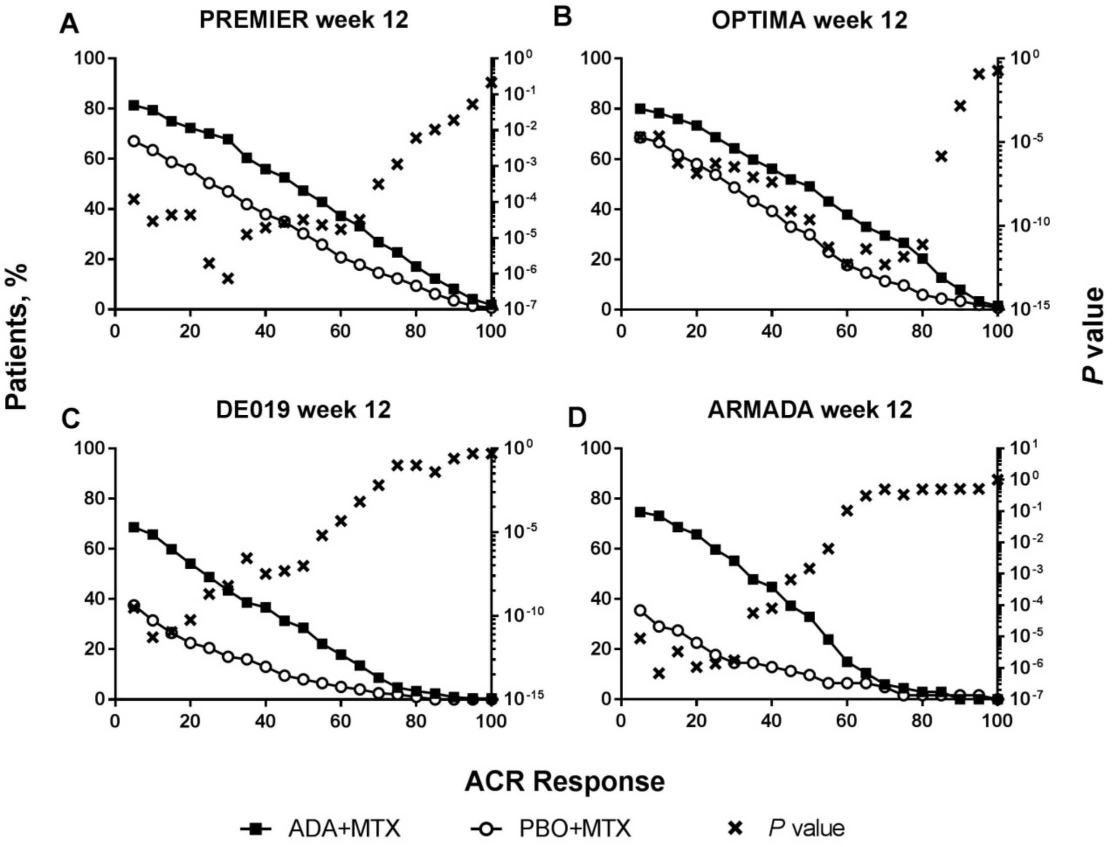

Supplemental Figure 2. Percent changes from baseline in CDAI scores in patients with early RA from (A) PREMIER and (B) OPTIMA and in patients with established RA from (C) DE019 and (D) ARMADA at week 12. *P* value of difference between response rates for patients treated with ADA+MTX and PBO+MTX. ADA, adalimumab; CDAI, Clinical Disease Activity Index; MTX, methotrexate; PBO, placebo; RA, rheumatoid arthritis.

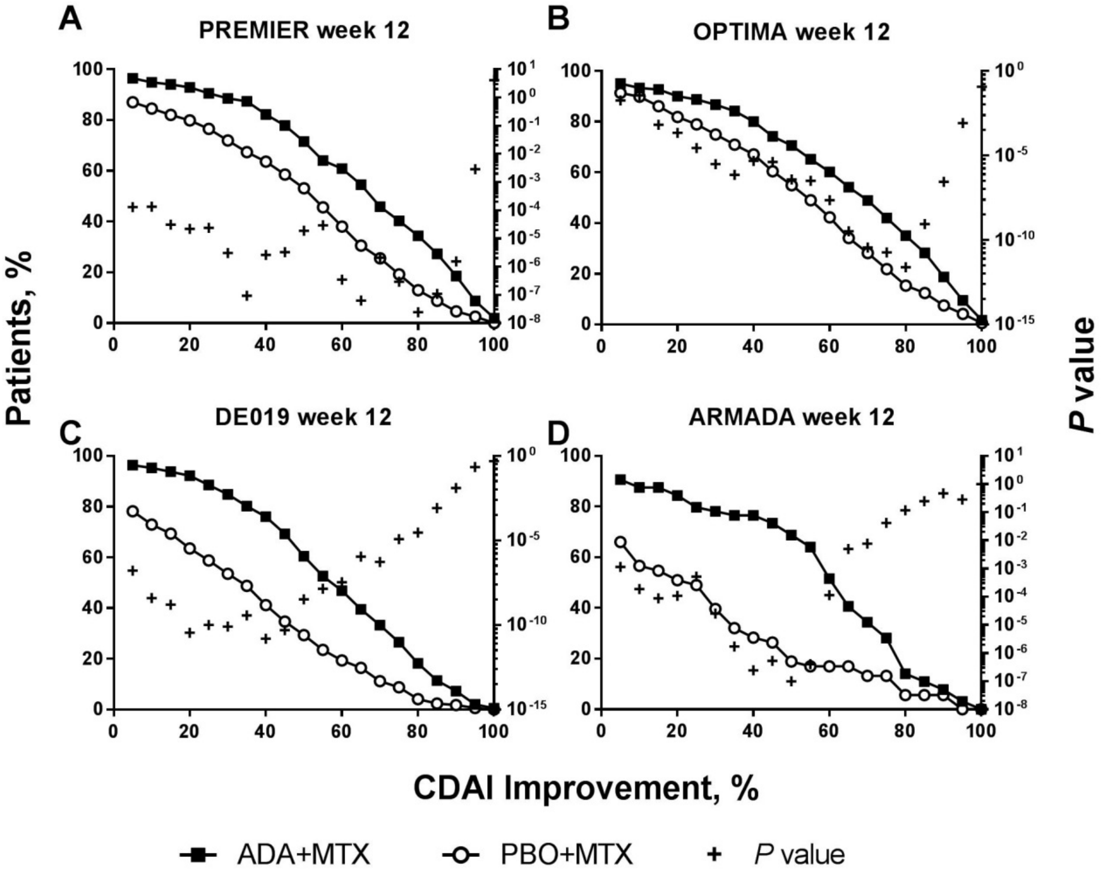

Supplemental Figure 3. Percent change from baseline in SDAI scores in patients with early RA from (A) PREMIER and (B) OPTIMA and in patients with established RA from (C) DE019 and (D) ARMADA at week 12. *P* value of difference between response rates for patients treated with ADA+MTX and PBO+MTX. ADA, adalimumab; MTX, methotrexate; PBO, placebo; RA, rheumatoid arthritis; SDAI, Simplified Disease Activity Index.

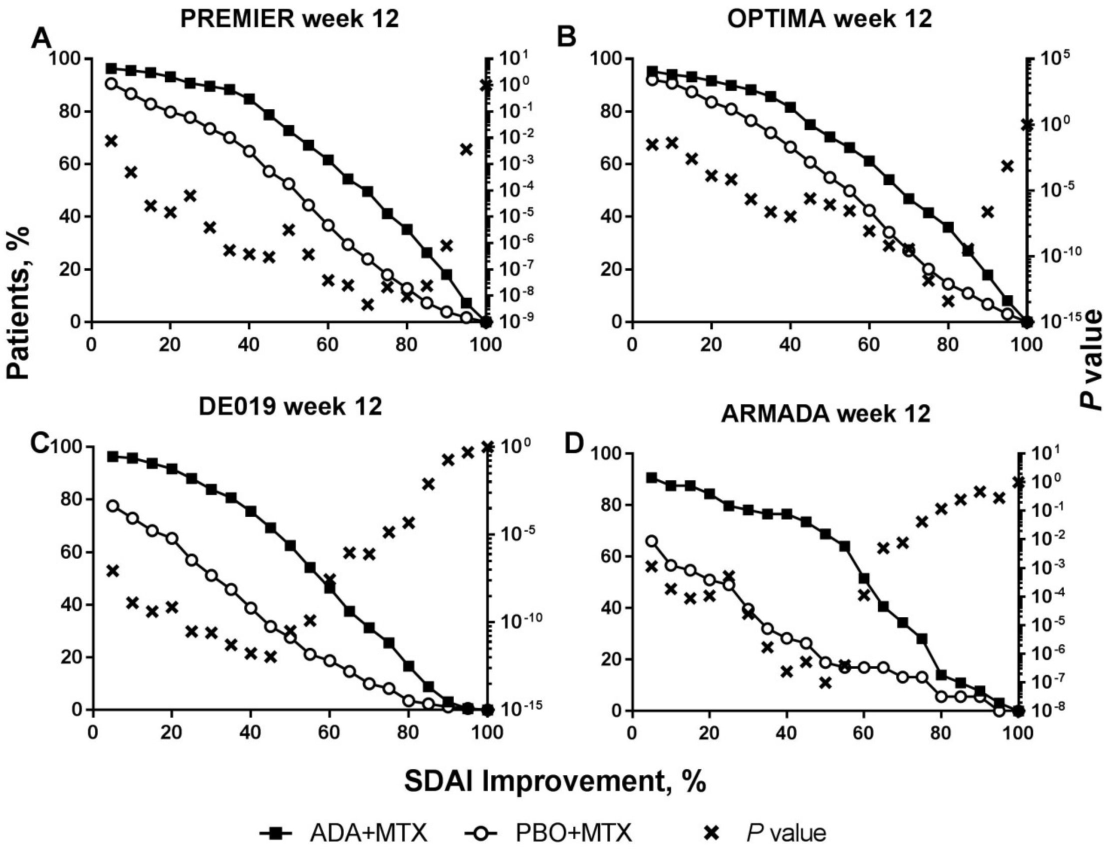

Supplemental Figure 4. Percent change from baseline in DAS28(CRP) scores in patients with early RA from (A) PREMIER and (B) OPTIMA and in patients with established RA from (C) DE019 and (D) ARMADA at week 12. *P* value of difference between response rates for patients treated with ADA+MTX and PBO+MTX. ADA, adalimumab; DAS28(CRP), 28-joint Disease Activity Score based on C-reactive protein; MTX, methotrexate; PBO, placebo; RA, rheumatoid arthritis.

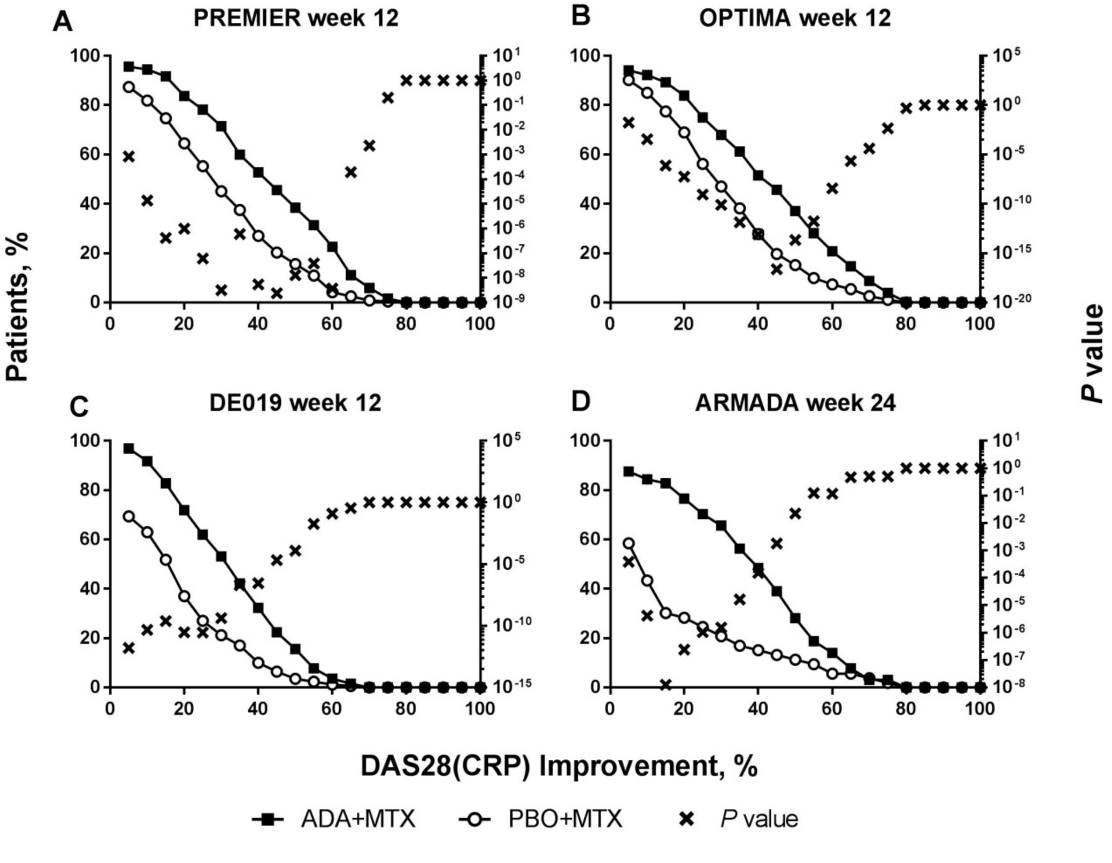

Supplement: Supplementary file 1 — Additional file 1: Table S1. Most discriminatory ACR, CDAI, SDAI and DAS28(CRP) cutoffs for each trial. Table S2. Most discriminatory CDAI cutoffs at week 24/26 for each trial in all patients and in subgroups of patients based on baseline CDAI and DAS28(CRP). Figure S1. ACR response rate in patients with early RA from (A) PREMIER and (B) OPTIMA and in patients with established RA from (C) DE019 and (D) ARMADA at week 12. P value of difference between response rates for patients treated with ADA+MTX and PBO+MTX. ADA, adalimumab; ACR, American College of Rheumatology; MTX, methotrexate; PBO, placebo; RA, rheumatoid arthritis. Figure S2. Percent change from baseline in CDAI scores in patients with early RA from (A) PREMIER and (B) OPTIMA and in patients with established RA from (C) DE019 and (D) ARMADA at week 12. P value of difference between response rates for patients treated with ADA+MTX and PBO+MTX. ADA, adalimumab; CDAI, Clinical Disease Activity Index; MTX, methotrexate; PBO, placebo; RA, rheumatoid arthritis. Figure S3. Percent change from baseline in SDAI scores in patients with early RA from (A) PREMIER and (B) OPTIMA and in patients with established RA from (C) DE019 and (D) ARMADA at week 12. P value of difference between response rates for patients treated with ADA+MTX and PBO+MTX. ADA, adalimumab; MTX, methotrexate; PBO, placebo; RA, rheumatoid arthritis; SDAI, Simplified Disease Activity Index. Figure S4. Percent change from baseline in DAS28(CRP) scores in patients with early RA from (A) PREMIER and (B) OPTIMA and in patients with established RA from (C) DE019 and (D) ARMADA at week 12. P value of difference between response rates for patients treated with ADA+MTX and PBO+MTX. ADA, adalimumab; DAS28(CRP), 28-joint Disease Activity Score based on C-reactive protein; MTX, methotrexate; PBO, placebo; RA, rheumatoid arthritis. [file 13075_2019_2005_MOESM1_ESM.pdf]
